# Prognostic Value of 5-ALA Fluorescence, Tumor Cell Infiltration and Angiogenesis in the Peritumoral Brain Tissue of Brain Metastases

**DOI:** 10.3390/cancers13040603

**Published:** 2021-02-03

**Authors:** Petra A. Mercea, Mario Mischkulnig, Barbara Kiesel, Lisa I. Wadiura, Thomas Roetzer, Romana Prihoda, Patricia Heicappell, Judith Kreminger, Julia Furtner, Adelheid Woehrer, Matthias Preusser, Karl Roessler, Anna S. Berghoff, Georg Widhalm

**Affiliations:** 1 Department of Neurosurgery, Medical University Vienna, Vienna General Hospital, Waehringer Guertel 18-20, 1090 Vienna, Austria; petra.mercea@meduniwien.ac.at (P.A.M.); mario.mischkulnig@meduniwien.ac.at (M.M.); barbara.kiesel@meduniwien.ac.at (B.K.); lisa.wadiura@meduniwien.ac.at (L.I.W.); romana.prihoda@gmail.com (R.P.); karl.roessler@meduniwien.ac.at (K.R.); georg.widhalm@meduniwien.ac.at (G.W.); 2 Division of Neuropathology and Neurochemistry, Department of Neurology, Medical University Vienna, Vienna General Hospital, Waehringer Guertel 18-20, 1090 Vienna, Austria; thomas.roetzer@meduniwien.ac.at (T.R.); adelheid.woehrer@meduniwien.ac.at (A.W.); 3 Division of Oncology, Department of Medicine I, Medical University Vienna, Vienna General Hospital, Waehringer Guertel 18-20, 1090 Vienna, Austria; patricia.heicappell@gmail.com (P.H.); judith.kreminger@meduniwien.ac.at (J.K.); matthias.preusser@meduniwien.ac.at (M.P.); 4 Department of Biomedical Imaging and Image-guided Therapy, Medical University Vienna, Vienna General Hospital, Waehringer Guertel 18-20, 1090 Vienna, Austria; julia.furtner@meduniwien.ac.at

**Keywords:** 5-ALA, brain metastases, peritumoral brain tissue, angiogenesis, local recurrence/progression

## Abstract

**Simple Summary:**

In a recent study, we observed 5-ALA fluorescence not only in brain metastases (BM) but also in the peritumoral brain tissue. However, the histopathological correlate of visible 5-ALA fluorescence in the peritumoral brain tissue is not fully understood. Therefore, we safely collected and analyzed tissue samples from fluorescing and non-fluorescing peritumoral brain tissue. Surprisingly, 5-ALA fluorescence in the peritumoral brain tissue did not correlate with tumor cell infiltration but did show a significant relation with angiogenesis. Moreover, the presence of angiogenesis significantly correlated with shorter time to local progression/recurrence and one-year survival. Consequently, angiogenesis in the peritumoral brain tissue might be a novel prognostic marker in BM. This represents the first study in the literature describing the prognostic impact of angiogenesis in fluorescent peritumoral brain tissue of BM, which might support individualized perioperative treatment concepts in the future.

**Abstract:**

Complete resection is an indispensable treatment option in the management of brain metastases (BM). 5-aminolevulinic acid (5-ALA) fluorescence is used for improved intraoperative visualization of tumor tissue in gliomas and was recently observed in BM. We investigated the potential of 5-ALA fluorescence to visualize the infiltrative growth of BM in the peritumoral brain tissue and its histopathological correlate. Patients with BM resection after 5-ALA administration and collection of tissue samples from peritumoral brain tissue were included. Each tissue sample was histopathologically investigated for tumor cell infiltration and angiogenesis. Altogether, 88 samples were collected from the peritumoral brain tissue in 58 BM of 55 patients. Visible 5-ALA fluorescence was found in 61 (69%) of the samples, tumor infiltration in 19 (22%) and angiogenesis in 13 (15%) of samples. Angiogenesis showed a significant correlation with presence of fluorescence (*p* = 0.008). Moreover, angiogenesis was related to visible 5-ALA fluorescence and showed an association with patient prognosis since it was significantly correlated to shorter time to local progression/recurrence (*p* = 0.001) and lower one-year survival (*p* = 0.031). Consequently, angiogenesis in the peritumoral brain tissue of BM might be a novel prognostic marker for individualized perioperative treatment concepts in the future.

## 1. Introduction

Brain metastases (BM) are a common and devastating complication in the clinical course of systemic malignancies [1,2]. These secondary brain tumors constitute the most frequent malignant neoplasms of the central nervous system with an increasing incidence rate [3]. Neurosurgical tumor resection is an important treatment option in the multimodal management of BM. The aim of surgery represents the complete and safe removal of detectable BM tissue. Nevertheless, incomplete resection of BM is not uncommon in the routine neurosurgical practice, resulting in high rates of local recurrence, even despite initiation of postoperative adjuvant therapies [4,5,6].

One major reason for such incomplete tumor resections might be insufficient detection of BM tissue during neurosurgical resection. Indeed, an unexpected residual tumor after surgery was identified by postoperative magnetic resonance imaging (MRI) in approximately 25% of BM [7,8]. Additionally, the peritumoral brain tissue of BM is assumed to be an important site for residual tumor tissue. In this sense, recent studies observed a high rate of BM with infiltrative growth into the peritumoral brain tissue although these tumors were initially considered as well-demarcated neoplasms [7,9,10]. Typically, this infiltrative behavior of BM consists either of a growth along pre-existing blood vessels in a so-called “vascular co-option” growth pattern or a diffuse “glioma-like” single cell infiltration of peritumoral brain tissue [9]. Due to evolving targeted therapies, angiogenesis of BM attracted more attention in the neurooncological field in the last few years [11,12]. However, the presence of angiogenesis in the peritumoral brain tissue and its potential impact on progression/recurrence of BM has not been clarified so far.

An innovative approach for improved intraoperative visualization of malignant brain tumor tissue is the application of 5-aminolevulinic acid (5-ALA) fluorescence [13,14]. Primarily, this technique was used for fluorescence-guided resections of high-grade gliomas [15]. Lately, visible 5-ALA fluorescence was observed in BM in the first patient series as well [16,17]. Although the majority of BM displayed a visible but heterogeneous 5-ALA fluorescence pattern, the impact of 5-ALA in BM still remains controversial [16,17]. Interestingly, visible 5-ALA fluorescence was recently also detected in the peritumoral brain tissue after BM resection in a subgroup of patients [16,17]. However, to date, the significance of this visible 5-ALA fluorescence has not been determined. If visible 5-ALA fluorescence could be used to identify tumor infiltration and/or angiogenesis in the peritumoral brain tissue, the technique might be useful for guidance of individualized perioperative treatment concepts.

The aim of this study was thus to analyze the histopathological correlate of visible 5-ALA fluorescence in the peritumoral brain tissue of BM and its influence on patient prognosis. For this purpose, we investigated specific histopathological parameters such as tumor infiltration and angiogenesis in a large series of tissue samples from fluorescing and non-fluorescing peritumoral tissue collected during resection of BM.

## 2. Results

In this study, 55 patients with a median age of 62 years (range: 27–82 years) with surgical BM resection after 5-ALA administration were included. At time of surgery, a single BM was present in 31 (56%) patients and multiple BM in 24 (44%) patients. In two patients more than one BM were removed in a single procedure and another patient was operated twice due to local tumor recurrence during the study period. At time of BM diagnosis, the primary tumor was known in 52 (95%) patients. Of these, lung cancer (*n* = 23/55; 42%) was the most common primary tumor type, followed by melanoma (*n* = 8/55; 14%) and breast cancer (*n* = 7/55; 13%). Further patient details are provided in Table 1.

### 2.1. Characteristics of BM

Altogether 58 BM were surgically removed in 55 patients during the study period. In 53 (91%) BM no previous surgery was performed, and 5 (9%) BM were local recurrences after previous resection. Of these five local recurrences after first surgery, initial postoperative treatment was radiotherapy of the resection cavity in three cases and whole-brain radiation therapy (WBRT) and stereotactic radiosurgery in one case. In the remaining patient, no postoperative radiotherapy was performed after surgery due to previous local radiation exposure with radiosurgery. The most common tumor localizations were the frontal lobe (*n* = 16/58; 28%), the cerebellum (*n* = 13/58; 22%) as well as the parietal lobe (*n* = 13/58; 22%). With regard to the contrast-enhancement (CE) pattern on MRI, heterogeneous CE was found in 27 (47%) BM, cystic CE in 25 (43%) BM and solid CE in 6 (10%) BM. Further details on BM are given in Table 2.

### 2.2. 5-ALA Fluorescence Characteristics of BM

**Figure 1:**
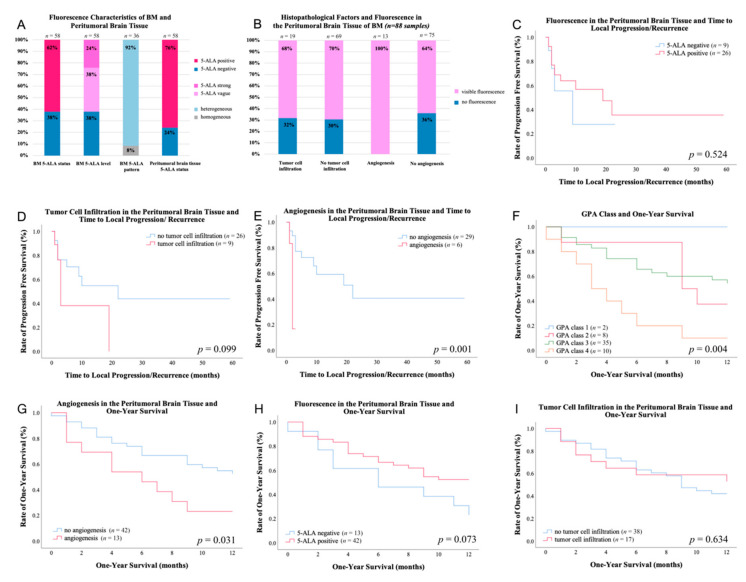
Illustration of intraoperative fluorescence characteristics in brain metastases/peritumoral brain tissue, histopathological correlate and postoperative follow-up. (**A**) 5-ALA fluorescence characteristics of the BM (fluorescence status, level and homogeneity) and the 5-ALA fluorescence status of the peritumoral brain tissue. (**B**) 5-ALA fluorescence status in samples (*n* = 88) from peritumoral brain tissue with presence or absence of tumor cell infiltration and angiogenesis. It is of note that angiogenesis was found only in tissue samples with visible 5-ALA fluorescence. (**C**–**E**) The Kaplan–Meier curves indicate the time to local progression/recurrence dependent on specific factors in the peritumoral brain tissue including (**C**) the 5-ALA fluorescence status, (**D**) tumor cell infiltration and (**E**) angiogenesis. It is of note that we found a significantly shorter median time to local progression/recurrence in brain metastases with presence of angiogenesis (2 months) compared to BM without angiogenesis in the peritumoral brain tissue (22 months; *p* = 0.001). (**F**–**I**) The Kaplan–Meier curves indicate the one-year survival rates dependent on (**F**) the GPA class as well as (**G**) angiogenesis in the peritumoral brain tissue, (**H**) the 5-ALA fluorescence status and (**I**) tumor cell infiltration in the peritumoral brain tissue. A significant correlation of the one-year survival with the GPA class was present (*p* = 0.004). Furthermore, we found a significantly lower one-year survival rate in patients with brain metastases with angiogenesis (23%) compared to absence of angiogenesis (52%; *p* = 0.031) in the peritumoral brain tissue.

Visible 5-ALA fluorescence was detected in 36 (62%) BM, whereas the remaining 22 (38%) tumors showed no fluorescence. Of fluorescing BM (*n* = 36), 22 (61%) BM demonstrated vague fluorescence and 14 (39%) BM strong fluorescence. In patients with multiple resected BM 5-ALA fluorescence of the tumors did not differ in intensity and heterogeneity. However, in one patient the peritumoral brain tissue of the first BM showed no visible fluorescence whereas the other BM presented with visible fluorescence (see Table S1). With regard to the fluorescence homogeneity, heterogeneous fluorescence was found in 33 (92%) BM and a homogeneous fluorescence pattern in 3 (8%) BM. See also Figure 1A. Visible 5-ALA fluorescence of the BM was significantly more common in BM with heterogenous CE on preoperative MRI (58%) compared to cystic CE (36%) and solid CE (6%; *p* = 0.047). However, no significant difference in the 5-ALA fluorescence status of the BM in relation to the tumor volume (*p* = 0.239), edema volume (*p* = 0.319) and primary tumor type (*p* = 0.497) was detected. Moreover, we could not find a significant correlation between administration of preoperative chemotherapy and 5-ALA fluorescence level (*p* = 0.776) and 5-ALA fluorescence status (*p* = 0.728) of the brain metastases as well as 5-ALA fluorescence level of the peritumoral brain tissue (*p* = 0.504). Additionally, there was no significant relation between local treatment of BM previous to surgery such as WBRT, SRS, radiation therapy and combinations of these in regard to 5-ALA fluorescence level (*p* = 0.891) and fluorescence status (*p* = 0.211) of the BM.

### 2.3. 5-ALA Fluorescence Characteristics of Peritumoral Brain Tissue

**Figure 2:**
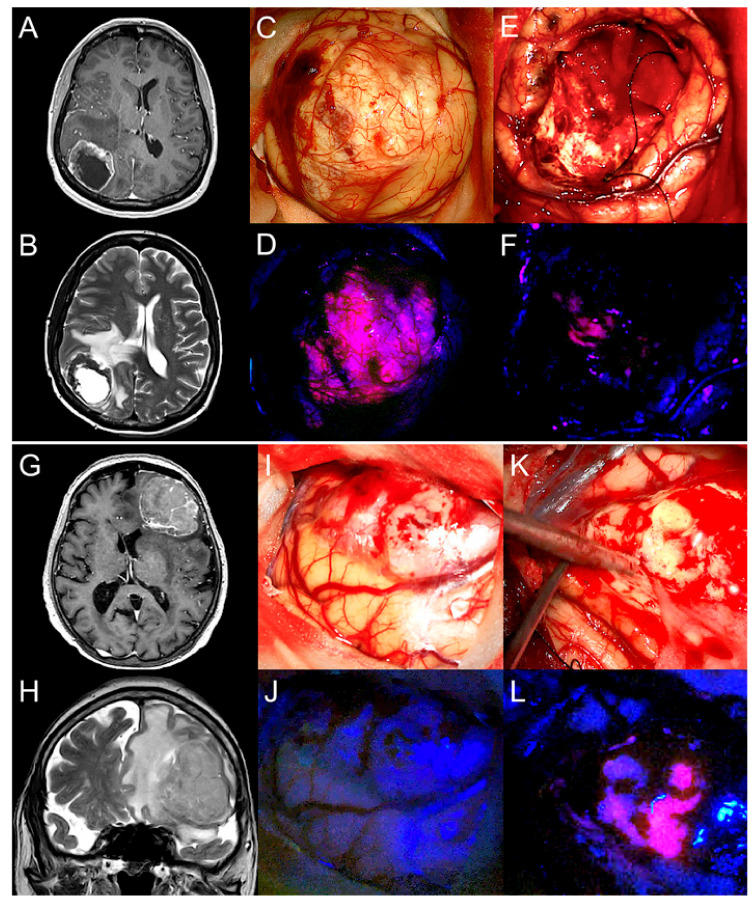
Characteristic fluorescence patterns of the tumor and peritumoral brain tissue in two patients with surgery of a brain metastasis after preoperative 5-ALA administration. (**A**) The brain metastasis of the first patient in the right occipital lobe demonstrates a cystic pattern of contrast-enhancement on T1-weighted MR images and (**B**) shows a distinct peritumoral edema on T2-weighted images. (**C**) After dura opening, the tumor is visible under conventional white-light microscopy and (**D**) demonstrates strong 5-ALA fluorescence under violet-blue excitation light. (**E**) Although the brain metastasis was completely removed under white-light microscopy, (**F**) visible fluorescence is detected in the peritumoral brain tissue. (**G**) The brain metastasis of the second patient in the left frontal lobe is characterized by heterogenous contrast-enhancement on T1-weighted images (**H**) with an extensive peritumoral edema. (**I**) During surgery, the brain metastasis is visible under conventional white-light microscopy, (**J**) but no visible fluorescence is detected under violet-blue excitation light. (**K**) After complete removal of the brain metastasis under white-light microscopy, (**L**) visible fluorescence is found in the peritumoral brain tissue although the brain metastasis itself did not reveal any visible fluorescence.

For each BM a representative tissue sample of the peritumoral brain tissue was chosen according to the 5-ALA fluorescence level intensity (positive/negative). Intraoperatively, 44 (76%) BM revealed visible 5-ALA fluorescence in the peritumoral brain tissue. Generally, this fluorescence effect was of vague appearance. In contrast, 13 (22%) BM did not show visible 5-ALA fluorescence in the peritumoral brain tissue. With regard to the fluorescence status of the BM, 30 (83%) of 36 tumors with visible 5-ALA fluorescence showed fluorescing peritumoral brain tissue (see Figure 2A–F). Despite the lack of visible 5-ALA fluorescence in 22 BM, 14 cases (64%) demonstrated visible 5-ALA fluorescence in the peritumoral brain tissue (see Figure 2G–L). Overall, we did not find a significant correlation between the 5-ALA fluorescence status of the BM and the peritumoral brain tissue (*p* = 0.092). Furthermore, no significant difference between visible 5-ALA fluorescence in the peritumoral brain tissue and the tumor volume (*p* = 0.663), CE pattern on MRI (*p* = 1.000), edema volume (*p* = 0.126) and primary tumor (*p* = 0.914) was found. Additionally, we did not find a significant relation between local preoperative treatment of BM such as WBRT, SRS, radiation therapy and combinations of these in regard to 5-ALA fluorescence level of the peritumoral brain tissue (*p* = 1.000).

### 2.4. Tissue Samples from Peritumoral Brain Tissue and Histopathology

At least one tissue sample from the peritumoral tissue was safely collected from all included BM (*n* = 58) and altogether 88 tissue samples (median: 1; range: 1–4 samples) were available for further histopathological analysis. The approximate volume of the biopsies from peritumoral brain tissue was 80–125 mm^3^. Visible 5-ALA fluorescence was present in 61 (69%) of these samples, whereas in the remaining 27 (31%) specimens no fluorescence was detected.

#### 2.4.1. Tumor Cell Infiltration

Based on one representative tissue sample from the peritumoral brain tissue of each BM, histopathological analysis revealed tumor infiltration in 18 (31%) of 58 BM with diffuse single cell infiltration in 13 (72%) and vascular co-option in 5 (28%) cases. With regard to the primary tumor comparing primarily lung cancers, melanomas, breast cancers and other tumor entities, we did not find a significant relation with the 5-ALA fluorescence status of the peritumoral brain tissue (*p* = 1.000), tumor cell infiltration (*p* = 0.516) and angiogenesis (*p* = 0.597). Further, we did not detect a significant association between the specific subtypes of lung cancer (non-small cell and small cell lung cancer) or breast cancer (luminal, human epidermal growth factor receptor 2 and triple negative) and 5-ALA fluorescence in the peritumoral brain tissue, tumor cell infiltration or angiogenesis (*p* = 1.000, respectively).

With regard to the 88 fluorescing and non-fluorescing samples from the peritumoral brain tissue, tumor cell infiltration was present in 19 (22%) of these specimens. Peritumoral brain tissue with visible 5-ALA fluorescence revealed tumor cell infiltration in 13 (21%) of 61 samples with diffuse single cell infiltration in 9 (69%) and vascular co-option in 4 (31%) cases. In the non-fluorescing samples of peritumoral brain tissue, tumor cells were present in 6 (22%) of 27 specimens with diffuse single cell infiltration in 5 (83%) cases and vascular co-option in one (17%) case. In detail, 19 (22%) samples presented with tumor cell infiltration out of which 13 (68%) presented with visible fluorescence. The remaining 69 (78%) samples with missing tumor cell infiltration showed visible fluorescence in 48 (64%) of cases (see Figure 1B). No significant relation between the 5-ALA fluorescence status of the peritumoral brain tissue and tumor cell infiltration (*p* = 1.000) was found.

#### 2.4.2. Angiogenesis

Histopathological analysis of the representative sample from the peritumoral brain tissue in each BM detected angiogenesis in 13 (22%) of 58 tumors. With regard to the 88 fluorescing and non-fluorescing samples, angiogenesis was observed in 13 (15%) of these specimens from the peritumoral brain tissue. Angiogenesis was significantly related to the 5-ALA fluorescence status of the peritumoral brain tissue and was only found in fluorescing brain samples (*n* = 13/61, 21%; *p* = 0.008). In contrast, angiogenesis was never detected in samples from non-fluorescing peritumoral brain tissue. In detail, from the samples with presence of angiogenesis (*n* = 13/88; 15%), all of them (*n* = 13/13; 100%) displayed visible fluorescence. The remaining samples (*n* = 75/88; 85%) with absent angiogenesis showed visible fluorescence in (70%) of cases (see Figure 1B). No significant relation between tumor cell infiltration and angiogenesis in the peritumoral brain tissue (*p* = 0.062) was observed.

### 2.5. Extent of Resection, Postoperative Course and Treatment

Postoperative MRI to assess the extent of resection (EOR) was performed in 33 (57%) of 58 BM showing a complete resection in 17 (52%) BM and incomplete resection in 8 (24%) BM. In 8 cases (24%), due to poor quality caused by motion artifacts, no clear statement on EOR according to postoperative MRI was possible. In the postoperative course, no significant side-effects related to 5-ALA fluorescence in BM were observed. At time of discharge, improved neurological status was found in 36 (66%) patients, 15 (27%) patients remained unchanged and 4 (7%) patients showed a postoperative neurological deterioration compared to the preoperative status. Of the four patients with neurological impairment, three patients showed improvement of symptoms, whereas one patient remained unchanged in the 3-month follow-up. With regard to the postoperative treatment after BM resection, local radiotherapy of the resection cavity was performed in 31 (56%) patients and whole-brain-radiation therapy in 17 (31%) patients. In seven patients (13%), no postoperative radiotherapy was indicated due to previous radiation exposure and high risk of radiation damage to vital brain parenchyma in five patients with recurrent BM as well as rapid progression of systemic disease and death within 4 weeks after surgery in two patients. Further, presence/absence of angiogenesis in the peritumoral brain tissue showed no significant correlation with local postoperative therapy (*p* = 1.000). Further details on postoperative treatment are given in Table 1.

### 2.6. Postoperative Follow-Up of BM

#### 2.6.1. Time to Local Progression/Recurrence or Distant Recurrence

**Figure 3:**
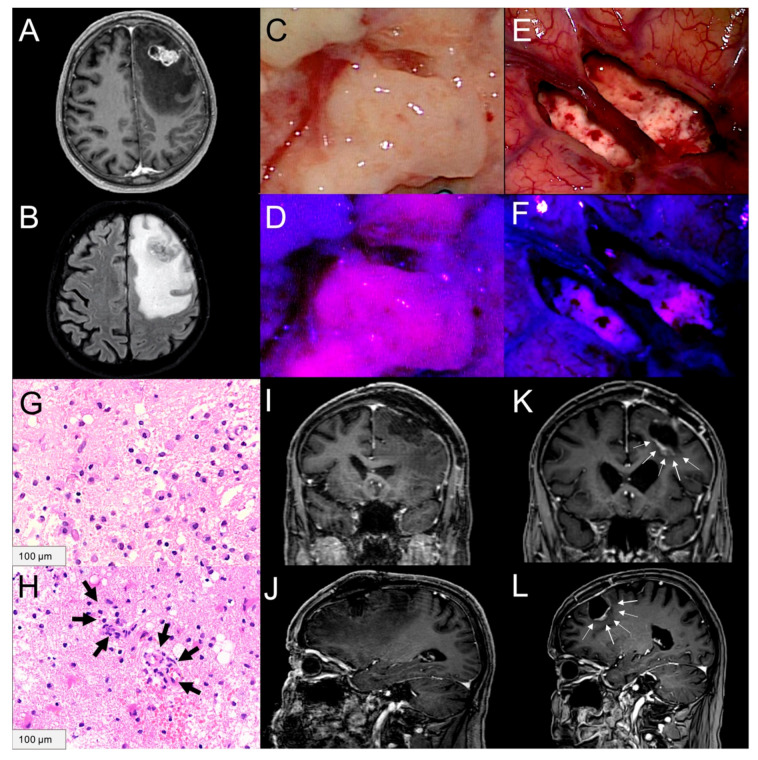
Illustration of fluorescence characteristics, corresponding tumor cell infiltration/angiogenesis and follow-up in a patient with known bladder cancer with surgery of a brain metastasis in the left frontal lobe after preoperative 5-ALA administration. (**A**) The brain metastasis shows heterogeneous contrast-enhancement on T1-weighted images (**B**) with a distinct peritumoral edema. (**C**) During surgery, the brain metastasis is visible under conventional white-light microscopy and (**D**) demonstrates vague 5-ALA fluorescence under violet-blue excitation light. (**E**) After complete tumor resection under white-light microscopy, (**F**) visible fluorescence is detected in the peritumoral brain tissue. (**G**) According to histopathological analysis no tumor cell infiltration is present in the peritumoral brain tissue, (**H**) but presence of angiogenesis (black arrows) is detected. (**I**) Postoperative MRI with coronal and (**J**) sagittal sequences confirms a complete resection without evidence of residual tumor. (**K**) In the first follow-up MRI a rapid tumor recurrence (white arrows) is detected on coronal and (**L**) sagittal sequences and the patient died 6 months after surgery.

In the present study, MRI follow-up controls were available in 35 patients. Our routine practice schedules the first follow-up MRI to be performed approximately 3 months after surgery. In four patients, the first follow-up MRI was conducted earlier (<2 months after surgery) due to development/deterioration of neurological symptoms. The median time to local progression/recurrence of BM was 19 months (range: 1–22 months; 95% median CI: 2.3–35.7) in the 35 BM with available MRI follow-up and did not significantly correlate with the 5-ALA fluorescence status of the BM (*p* = 0.826) or the peritumoral brain tissue (*p* = 0.524, Figure 1C) and tumor cell infiltration (*p* = 0.099, Figure 1D) in the peritumoral brain tissue. However, we found a significantly shorter median time to local progression/recurrence in BM with presence of angiogenesis (2 months) compared to BM without angiogenesis in the peritumoral brain tissue (22 months; *p* = 0.001, Figure 1E). Further, the median time to distant recurrence within the brain was 10 months (range: 1–46 months; median 95% CI: 0.0–21.9). No significant association between the median time to distant recurrence and the 5-ALA fluorescence status of the BM (*p* = 0.506) or peritumoral brain tissue (*p* = 0.060), tumor infiltration (*p* = 0.587) or angiogenesis (*p* = 0.354) in the peritumoral brain tissue was found. An illustrative case is shown in Figure 3A–L. In univariate analysis, we found that presence of angiogenesis in the peritumoral brain tissue was a significant hazard for a shorter progression free survival (hazard ratio (HR): 5.924; 95%-CI: 1.709–20.451; *p* = 0.005; Cox regression model). In contrast, local postoperative radiation treatment showed no significant influence regarding time to local progression/recurrence (hazard ratio (HR): 1.249; 95%-CI: 0.275–5.675; *p* = 0.774; Cox regression model) as well as WBRT (hazard ratio (HR): 2.224; 95%-CI: 0.350–14.150; *p* = 0.397; Cox regression model). Accordingly, in multivariate analysis angiogenesis remained a significant hazard for shorter progression free survival (hazard ratio (HR): 5.607; 95%-CI: 1.517–20.717; *p* = 0.010; Cox regression model) compared to local postoperative radiation treatment [hazard ratio (HR): 1.008; 95%-CI: 0.214– 4.746; *p* = 0.992; Cox regression model] and WBRT (hazard ratio (HR): 1.232; 95%-CI: 0.170–9.942; *p* = 0.837; Cox regression model). Details are presented in Table S2.

#### 2.6.2. Survival Rate

We found a significant correlation of one-year survival with the GPA class (*p* = 0.004, Figure 1F). Furthermore, a significantly lower one-year survival rate was observed in patients suffering from BM with angiogenesis (3 of 13; 23%; mean 95% CI: 3.8–8.3) compared to absence of angiogenesis (22 of 42; 52%; mean 95% CI: 7.5–10.1; *p* = 0.031, Figure 1G) in the peritumoral brain tissue. However, we found no significant association between one-year survival and visible 5-ALA fluorescence (*p* = 0.073, Figure 1H) or tumor cell infiltration (*p* = 0.634, Figure 1I) in the peritumoral brain tissue. In univariate analysis, presence of angiogenesis was a significant hazard for a decrease in one-year survival (hazard ratio (HR): 2.228; 95%-CI: 1.035–4.793; *p* = 0.040; Cox regression model) as well as the GPA score (HR: 2.162; 95%-CI: 1.165–4.043; *p* = 0.016; Cox regression model). We found that local postoperative radiation treatment (HR: 0.782; 95%-CI: 0.308–2.429; *p* = 0.782; Cox regression model) and WBRT (HR: 1.619; 95%-CI: 0.559–4.693; *p* = 0.375; Cox regression model) had no significant influence on one-year survival as well as postoperative chemotherapy (HR: 0.943; 95%-CI: 0.442–1.959; *p* = 0.875; Cox regression model). According to multivariate analysis, angiogenesis in the peritumoral brain tissue (HR: 2.629; 95%-CI: 1.144–6.038; *p* = 0.023; Cox regression model) and GPA class (HR: 2.389; 95%-CI: 1.280–4.456; *p* = 0.006; Cox regression model) remained significant as independent prognostic factors regarding one-year survival. In contrast, local postoperative radiation treatment (HR: 0.741; 95%-CI: 0.246–2.227; *p* = 0.593; Cox regression model), WBRT (HR: 1.275; 95%-CI: 0.408–3.986; *p* = 0.677; Cox regression model) and postoperative chemotherapy (HR: 1.156; 95%-CI: 0.529–2.523; *p* = 0.716; Cox regression model) were no significant factors influencing one-year survival. Details are shown in Table S2.

## 3. Discussion

Tumor cell infiltration of the peritumoral brain tissue is a common finding in BM that might result in local recurrence and thus worse patient prognosis [9,18]. However, intraoperative visualization of such tumor cell infiltration is still challenging. Two decades ago, the 5-ALA fluorescence technique was introduced as an innovative method for improved visualization of tumor tissue during surgery of high-grade gliomas and is nowadays applied to safely increase the EOR at many neurosurgical centers worldwide [13,14,15]. In the largest series to date, we found visible 5-ALA fluorescence also in 66% of 157 BM [17]. Interestingly, we observed visible 5-ALA fluorescence also in the peritumoral brain tissue after assumed complete BM resection in a subgroup of patients [17]. Thus, we speculated that 5-ALA fluorescence might visualize tumor cell infiltration in adjacent brain tissue of BM and collected 88 fluorescing and non-fluorescing samples from this region for histopathological analysis.

In this study, we found visible 5-ALA fluorescence in the peritumoral brain tissue during BM surgery in 76% of cases. Surprisingly, preoperative chemotherapy of the primary tumor and local radiation treatment preceding the resection of BM did not show a significant correlation with 5-ALA fluorescence, neither in the BM nor in the peritumoral brain tissue. We found tumor cell infiltration of the peritumoral brain tissue in 31% of BM. According to the data, we did not find a significant correlation between the 5-ALA fluorescence status of the peritumoral brain tissue and tumor cell infiltration. However, this might be influenced by a selection bias, since the number of samples taken from the peritumoral brain tissue was low (median 1; range: 1–4) and identification of tumor cell infiltration could simply be missed. Nonetheless, similar to our study, Kamp et al. found tumor cells in only 33% of specimens from the resection cavity of BM with visible 5-ALA fluorescence [16]. It is of note that a large portion of cases demonstrated visible 5-ALA fluorescence in the peritumoral brain tissue, albeit the BM itself did not show any visible 5-ALA fluorescence. Therefore, we assume that visible 5-ALA fluorescence in the peritumoral brain tissue of BM is not primarily caused by tumor cell infiltration. The lack of correlation between visible 5-ALA fluorescence in the peritumoral brain tissue and presence of tumor tissue might be induced by diffuse leakage of protoporphyrin IX, the fluorescing substrate within the 5-ALA metabolism, into the adjacent brain tissue. Similarly, Utsuki et al. discussed the “false positive” effect of 5-ALA fluorescence in malignant brain tumors [19]. Namely, the authors detected immune-cell infiltration and reactive astrocytes in fluorescent areas of peritumoral edema rather than tumor cell infiltration [19]. Additionally, Siam et al. found unique glial activation, a “glial-defense system”, at the tumor-brain-interface [18]. We assume, that these important co-factors might additionally influence visible 5-ALA fluorescence in the peritumoral brain tissue, which challenges the current understanding of the mechanism of 5-ALA fluorescence. Therefore, in the scope of a future study, we intend to address these crucial points and investigate immune-cell infiltration in fluorescing peritumoral brain tissue of BM. Nevertheless, a recent study found a significant correlation between 5-ALA fluorescence in the peritumoral brain tissue and tumor cell infiltration [20]. However, the authors relativized their findings since visible fluorescence was also found in peritumoral brain tissue with only very scarce tumor cell infiltration [20].

Further, in our study, we did not observe a significant association of visible 5-ALA fluorescence in the peritumoral brain tissue or tumor cell infiltration and time to progression/recurrence or one-year survival. In the literature, only reports correlating the 5-ALA fluorescence status of BM tumor bulk with prognostic factors are available. In this sense, Kamp et al. assumed in two of their studies that the 5-ALA fluorescence status in the tumor tissue of BM might be considered as a prognostic marker with a more aggressive behavior in BM along with absence of fluorescence [21,22]. Siam et al. described a significantly lower survival rate in patients with tumor infiltrated peritumoral brain tissue compared to patients without tumor infiltration [18]. However, the conflicting findings between the study of Siam et al. [18] and our study might be related to tissue sampling differences. In contrast to our study, Siam et al. [18]. included only BM with non-eloquent localization; thus, a higher number of samples from the peritumoral brain tissue (median: three samples per patient) could be collected compared to our study including also BM with eloquent localization (median: one sample per patient). By collecting a higher number of samples per BM, the likelihood was increased to detect tumor cell infiltration in at least one of the collected specimens [18]. We suspect a regional heterogeneity of tumor cell infiltration in brain tissue adjacent to BM, which has to be further investigated in future studies, especially in view of patient prognosis.

Surprisingly, angiogenesis was significantly related to the 5-ALA fluorescence status in our study since it was only found in fluorescing samples of peritumoral brain tissue. In line, a previous study of Roberts et al. in glioblastoma patients also suggested that approximately 4% of the specimens with visible 5-ALA fluorescence were either fully necrotic or showed abnormal, prominent vasculature [23]. It is of note that we also observed a significantly shorter time to local progression/recurrence (2 vs. 22 months) and lower one-year survival rate (23% vs. 52% one-year survival) in BM with angiogenesis in the peritumoral brain tissue compared to BM without angiogenesis in the adjacent brain tissue. To our best knowledge, the association of 5-ALA fluorescence in the peritumoral brain tissue of BM and presence of angiogenesis as well as the negative impact of angiogenesis on the time to progression/recurrence and one-year survival has not been described so far. Biologically, the observed angiogenesis might represent tumor induced preparation of the peritumoral brain tissue to allow vascular co-optive growth [9,24]. Indeed, the vascular co-optive growth pattern was frequently observed in autopsy specimens of BM [9,24]. Since visible 5-ALA fluorescence significantly correlated with angiogenesis, the intraoperative 5-ALA fluorescence status of the peritumoral brain tissue in BM might guide individualized perioperative treatment concepts. Moreover, due to the fact that angiogenesis was never found in non-fluorescing brain tissue in our study, lack of 5-ALA fluorescence in the peritumoral brain tissue might be an intraoperative indicator for absence of angiogenetic features. In contrast, angiogenesis might be present in case of visible 5-ALA fluorescence and, thus, a supramaximal resection with additional safe removal of a “safety margin” of 5 mm from the peritumoral brain tissue could be considered especially in BM with non-eloquent localization to improve the patient prognosis [25]. In this sense, Yoo et al. demonstrated an improved local tumor control and a significantly improved two-year survival in comparison to complete resection of the BM alone (63% vs. 29% two-year survival) [25]. In case of eloquent BM localization and non-resectable peritumoral brain tissue with biopsy confirmed or suspicion of angiogenesis a modification of the postoperative radiotherapy plan with extension of the radiation field around the resection cavity might be considered. However, angiogenesis was absent in 48/61 (79%) fluorescent samples of the peritumoral brain tissue. This might also explain the non-significant correlation between fluorescence in peritumoral brain tissue and one-year survival. Nevertheless, postoperative treatment such as local radiation therapy and WBRT did not show a significant influence on time to local progression/recurrence, whereas angiogenesis remained an independent factor even in multivariate analysis. According to univariate and multivariate analyses, postoperative treatment (local radiation therapy and WBRT) as well as adjuvant chemotherapy did not have any significant effect on one-year survival compared to angiogenesis and GPA class, which remained independent factors in multivariate analysis. The association of the standard prognostic score GPA (graded prognostic assessments) underscores that we investigated a regular BM cohort with a certain range of prognostic parameters. Altogether, future studies with a larger number of BM are needed to confirm our preliminary results and extensively investigate the significance of angiogenesis/fluorescence in the peritumoral brain tissue of BM.

Although our study is among the first to investigate the association of 5-ALA fluorescence with tumor cell infiltration and angiogenesis in BM, we have to interpret the results in the light of some limitations. In this sense, we were able to include only a fraction of patients treated with resection of BM as collection of tissue samples of the peritumoral brain tissue was not feasible in all patients especially due to tumor localization in highly eloquent areas. We analyzed one full section of each BM and specimens of peritumoral brain tissue. As serial sectioning was not performed, different patterns of infiltration and angiogenesis throughout the tumor sample cannot entirely be ruled out. In our view, the volume of the biopsies (approximately 80–125 mm^3^) of the single section is sufficient to be considered as representative of the total volume of peritumoral brain tissue. However, it cannot definitely be excluded that specific pathological features leading to 5-ALA fluorescence (i.e., angiogenesis, tumor cell infiltration, or other) are missed in this single section. Moreover, the number of samples taken from the peritumoral brain tissue was limited (median 1; range: 1–4) and identification of tumor cell infiltration could be missed. Consequently, we have to interpret out results with caution. Nevertheless, although the limited number of samples certainly might cause a bias, the current study is, to our best knowledge, the first to systemically explore angiogenesis in the peritumoral area of resected BM. Additionally, one must consider that, in certain cases, peritumoral brain tissue of BM includes functional brain tissue and, thus, cannot be biopsied without harming the patient. Another limitation is that the rate of samples from peritumoral brain tissue in our study might be lower in cases with a poor margin. Therefore, our strategy of tissue collection only from safe sampling localizations might have systematically biased against cases with a poor margin. Thus, we suggest that our findings regarding angiogenesis and patient prognosis should be regarded carefully. Additionally, our patient cohort is quite small; therefore we assume that analysis including a larger patient cohort might show differing results. Even more, if more extensive tissue sampling and serial sectioning is performed. Therefore, we suggest, that our observed preliminary findings and certainly the impact on the future treatment of BM patients need to be investigated in larger clinical trials.

## 4. Materials and Methods

Adult patients (≥18 years) with neurosurgical resection of BM after preoperative 5-ALA administration at the Department of Neurosurgery, Medical University Vienna, were included. The utilization of 5-ALA for the resection of BM was determined by the individual treating neurosurgeon. 5-ALA was not administered in patients with BM who required urgent surgery due to acute mass effect and/or hydrocephalus. At our institution, a mandatory precondition for elective resection of BM is the presence of a stable primary disease confirmed by the attending oncologist. Additionally, patients with known contraindications to 5-ALA were not included in this study. Patients were only included after giving informed consent to the participation in the present study. Furthermore, only BM were included in this study after histopathological confirmation. Brain tumors other than BM were excluded. Finally, only BM with available tissue samples from the peritumoral brain tissue with known 5-ALA fluorescence status were included. Our study was approved by the local ethics committee of the Medical University Vienna (EK: 419/2009; Amendment).

### 4.1. Preoperative Imaging and Clinical Data

The pattern of contrast enhancement (CE) on preoperative MRI was classified as “cystic”, “solid” and “heterogeneous” by an experienced neuroradiologist (J.F.). Quantification of the volume of the tumor and peritumoral edema was determined by volumetric measurements with a specialized software (Brainlab Elements SmartBrush, Version 2.6, Brainlab AG). Moreover, the localization of the resected BM as well as the number of BM (single or multiple) at time of surgery were determined. The Graded Prognostic Assessment (GPA) class as a prognostic index for patients with BM and clinical data were retrieved from the hospital archive system (AKIM) and the Vienna Brain Metastasis Registry.

### 4.2. 5-ALA Management and Neurosurgical Resection of BM

A standard dosage of 5-ALA (20 mg/kg bodyweight) was administered orally approximately three hours prior to anesthesia. For visualization of potential 5-ALA fluorescence a modified surgical microscope (NC4/Pentero, Carl Zeiss Surgical GmbH, Oberkochen, Germany) was applied. All patients underwent neurosurgical resection of BM with assistance of a neuronavigation system for intraoperative guidance. For maximum safe BM resection in eloquent brain areas, navigation with diffusion tensor imaging/functional MRI and/or intraoperative monitoring including cortical/subcortical mapping and stimulation was used to minimize the risk of a new postoperative neurological deficit. During surgery, the visible fluorescence status (visible or no fluorescence), fluorescence level (strong, vague or negative) and fluorescence homogeneity (homogeneous or heterogeneous) of each resected BM was determined by the performing neurosurgeon as described previously [17]. Generally, tissue sampling from the peritumoral brain tissue was conducted after assumed complete resection of the BM. For tissue sampling we usually applied a biopsy forceps with a diameter of the tip of 5 mm (Aesculap^®^ microform, FD216R). Tissue samples from the peritumoral brain tissue were safely collected only in localizations without the risk of causing postoperative neurological deterioration. The site of tissue collection was primarily based on the fluorescence status of the peritumoral brain tissue and the safety of tissue collection. Thus, tissue sampling did not usually depend on macroscopic factors such as suspicious vessels in the peritumoral brain tissue or suspected infiltrated white matter. In detail, the resection cavity was checked for potential 5-ALA fluorescence in the peritumoral brain tissue after assumed complete BM removal. In case of visible fluorescence, tissue samples were safely collected from fluorescing regions. In case of absence of visible fluorescence, tissue sampling from the peritumoral brain tissue was performed from localizations with maximum possible distance from eloquent brain areas. The 5-ALA fluorescence status (visible or no fluorescence) was documented for each tissue sample from the peritumoral brain tissue. Due to potential occurrence of significant brain-shift following tumor removal, we did not systematically use image guidance with neuronavigation for precise localization of the peritumoral brain tissue. Patients were finally included in the current study if at least one tissue sample of the peritumoral brain tissue could be safely collected during BM surgery.

### 4.3. Histopathological Assessment

**Figure 4:**
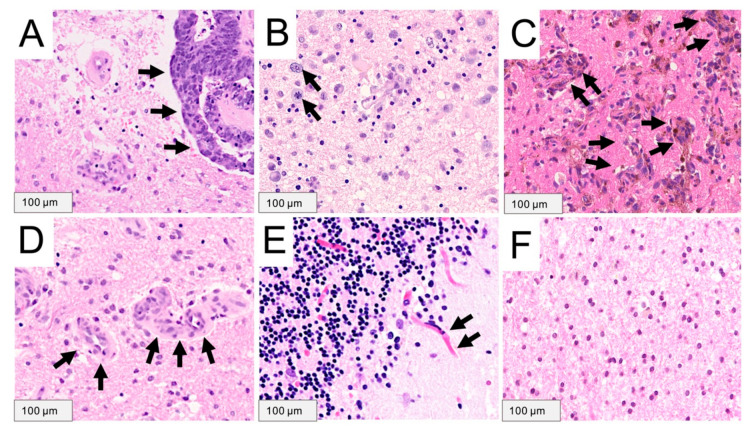
Histopathological examples demonstrating the different tumor cell infiltration patterns and angiogenetic features in the peritumoral brain tissue of brain metastases using hematoxylin and eosin stains under light-microscopy. (**A**) A well-demarcated growth pattern is present in a patient with a brain metastasis (black arrows) from colorectal cancer without evidence of tumor cell infiltration in the peritumoral brain tissue. (**B**) In contrast, histopathological assessment demonstrates tumor cell infiltration in the peritumoral brain tissue with either a diffuse “glioma-like” single cell infiltration (black arrows) or (**C**) growth along pre-existing blood vessels in a “vascular co-option” growth pattern (black arrows) in two patients with known melanoma. (**D**) Further, histopathological analysis shows presence of angiogenesis with a characteristic multilayered endothelium (black arrows) in the peritumoral brain tissue in a patient with colorectal cancer. (**E**) Vessels with a single endothelial layer were defined as “no angiogenesis”, (**F**) whereas angiogenesis is absent in another patient with non-small cell lung cancer.

A board-certified neuropathologist verified the diagnosis of BM. Additionally, all collected tissue samples from the peritumoral brain tissue were further investigated. For this purpose, presence of tumor cells, different infiltration patterns and angiogenesis were evaluated on hematoxylin and eosin stains on one section per sample. The infiltrative tumor growth pattern was classified as either diffuse single cell infiltration or vascular co-option as described previously [9]. Moreover, presence of angiogenesis in the peritumoral brain tissue was defined as vessels presenting with multilayered endothelia as previously determined [26]. As outlined in Figure 4A–F vessels with a single endothelial layer were defined as “no angiogenesis” while presence of a multiple endothelial cell layer as well as diversity in vessel configuration was defined as angiogenesis. If more than one sample was available per tumor, one representative specimen was selected to indicate the status of tumor cell infiltration or angiogenesis of each BM. Thus, tumor cell infiltration or angiogenesis was considered to be present in the peritumoral brain tissue, if at least one sample showed histopathological features of tumor tissue or angiogenesis. The different growth patterns and angiogenesis are illustrated in Figure 4.

### 4.4. Postoperative Course

After surgery, patients were protected from strong light sources to avoid potential 5-ALA related phototoxic side effects for a minimum time period of 24 h. Routinely, a postoperative computerized tomography of the brain was performed on the first postoperative day to exclude intracranial hematoma or ischemia. In the last years, postoperative MRI preferentially within 72 h after surgery was implemented into our standards of perioperative patient management in BM to assess their EOR. The postoperative neurological course at discharge was compared to the preoperative neurological status and classified as unchanged, improved or deteriorated. In patients with postoperative neurological impairment, the symptoms were reevaluated for potential improvement in the 3 months follow-up visit. In each patient, postoperative treatment was individually determined by an interdisciplinary tumor board. Radiotherapy was performed according to standard protocols used by our Department of Radiooncology. Therefore, supramarginal radiotherapy of the resection cavity was performed with a total of 35 Gy and 5 Gy in each fraction. SRS was either performed with lineac or gamma knife radiation. Hippocampal sparing WBRT was not applied in the present series. For WBRT, 10 fractions of 3 Gy (total 30 Gy) were applied. Data on time to local progression of a residual tumor, local recurrence after complete resection in the region of the surgically treated BM or distant recurrence in the brain at another site than the surgically removed BM were retrieved from regular follow-up MRI assessed by the experienced neuroradiologist (J.F.) whenever available. The data on one-year survival were obtained from the Vienna Brain Metastasis Registry.

### 4.5. Statistical Analyses

All statistical analyses were performed with SPSS 26.0 software (SPSS Inc., Chicago, IL, USA). Inferential analyses were used as appropriate by the use of Chi^2^-tests, Fisher’s-exact test and the Mann–Whitney U test for assessment of differences in 5-ALA fluorescence status of BM or peritumoral brain tissue with the volume of tumor/edema, primary tumor type and CE on preoperative MRI. Furthermore, differences between 5-ALA fluorescence in the collected tissue samples and tumor cell infiltration, growth pattern and angiogenesis were investigated. Due to the exploratory and hypothesis generating design of the present study no adjustment for multiple testing was applied [20]. The primary endpoint in our patient cohort was determined as the one-year survival time, which was defined as the time from diagnosis of BM to death within the first 12 months after surgery. Kaplan–Meier curves were used to estimate the one-year survival and the log-rank test was applied to investigate group differences. For statistical analyses we grouped the different postoperative treatments into (1) no postoperative treatment, (2) local radiation treatment (SRS and radiotherapy of resection cavity) and (3) WBRT. Further, univariate analyses were performed according to a Cox regression hazards model. Variables with significant univariate results were entered into a multivariable Cox regression hazards model. A *p*-value < 0.05 was considered as statistically significant.

## 5. Conclusions

In this study, we descriptively analyzed visible 5-ALA fluorescence, tumor cell infiltration and angiogenesis in the peritumoral brain tissue of BM. Further, we investigated the influence of these factors on patient prognosis. According to our data, 5-ALA fluorescence in the peritumoral brain tissue did not show an association with tumor cell infiltration and neither the 5-ALA fluorescence status nor tumor cell infiltration significantly correlated with patient prognosis. However, we found a significant relation of angiogenesis in the peritumoral brain tissue with visible 5-ALA fluorescence as well as with the time to progression/recurrence and the one-year survival. Nevertheless, due to the heterogeneity of our patient population and heterogeneous cohort of brain metastases further analyses will be necessary to provide a basis for future surgical treatment options such as supramarginal resection. So far, our data indicate that angiogenesis in the peritumoral brain tissue might be a novel prognostic marker in BM and 5-ALA fluorescence could support guiding individualized perioperative treatment concepts in future.

## Figures and Tables

**Table 1:** Patients characteristics.

		*n*	%
Number of patients		55	(100)
Gender	male:female	1:1.04	
Age	median (range)	62 years (27–82)	
GPA class	1	2	(4)
	2	8	(15)
	3	35	(64)
	4	10	(18)
Number of BM per patient			
	single	31	(56)
	multiple	24	(44)
Extracranial metastases			
	yes	31	(56)
	no	24	(44)
Primary tumor			
lung cancer		23	(42)
NSCLC		22	(96)
SCLC		1	(4)
melanoma		8	(14)
breast cancer		7	(13)
luminal		2	(29)
Her2		2	(29)
triple negative		2	(29)
unknown		1	(13)
colorectal		5	(9)
renal cell cancer		3	(5)
ovarian cancer		1	(2)
cervical cancer		1	(2)
gastric cancer		1	(2)
testicular cancer		1	(2)
urothelium cancer		1	(2)
sarcoma		1	(2)
cancer of unknown origin		3	(5)
Treatment before BM resection			
Chemotherapy			
	yes	33	(60)
	no	21	(38)
	n.d.	1	(2)
Previous local treatment of BM			
No previous local therapy		45	(82)
Primary resection only		1	(2)
Surgery + local radiation therapy		4	(7)
SRS		1	(2)
WBRT		1	(2)
WBRT + local radiation therapy		2	(4)
n.d.		1	(2)
Postoperative KPS	median (range)	80 (30–100)	

BM—brain metastases, GPA—graded prognostic assessment, HER2—human epidermal growth factor receptor 2, KPS—Karnofsky Performance Score, NSCLC—non small cell lung cancer, SCLC—small cell lung cancer, SRS—stereotactic radiosurgery, WBRT—whole-brain radiation therapy.

**Table 2:** Characteristics of brain metastases.

	*n*	%
Number of resected BM	58	(100)
Primary resection of BM		
yes	53	(91)
no	5	(9)
Tumor volume		
median (range)	13.7 cm^3^ (0.4–60.5)	
Localization		
frontal	16	(28)
cerebellar	13	(22)
parietal	13	(22)
temporal	7	(12)
occipital	7	(12)
central	2	(3)
CE pattern on MRI		
heterogeneous	27	(47)
cystic	25	(43)
solid	6	(10)
Volume of edema		
median (range)	45.2 cm^3^	(3.9–211)
5-ALA fluorescence in BM		
visible fluorescence	36	(62)
vague	22	(61)
strong	14	(39)
no fluorescence	22	(38)
5-ALA homogeneity		
heterogeneous	33	(92)
homogeneous	3	(8)
5-ALA fluorescence in peritumoral brain tissue		
positive	44	(76)
negative	14	(24)
Peritumoral brain tissue—characteristics of tissue samples		
Number of samples	88	(100)
5-ALA fluorescence status		
positive	61	(69)
negative	27	(31)
Tumor cell infiltration		
present	19	(22)
diffuse single cell infiltration	14	(74)
vascular co-option	5	(26)
absent	69	(78)
Angiogenesis		
present	13	(15)
absent	75	(85)

## Data Availability

The data presented in this study are available on request from the corresponding author. The data are not publicly available due to privacy restrictions since they contain information that could compromise the privacy of the study participants.

## Supplementary Materials

The following are available online at https://www.mdpi.com/2072-6694/13/4/603/s1, Table S1: Characteristics of multiple resected brain metastases, Table S2: Univariate and multivariate analyses.

## References

[1] Nayak L., Lee E.Q., Wen P.Y. (2012). Epidemiology of Brain Metastases. Curr. Oncol. Rep..

[2] Weinberg J., Lang F.F., Sawaya R. (2001). Surgical management of brain metastases. Curr. Oncol. Rep..

[3] Gavrilovic I.T., Posner J.B. (2005). Brain metastases: Epidemiology and pathophysiology. J Neurooncol..

[4] Kocher M., Soffietti R., Abacioglu U.M., Villà S., Fauchon F., Baumert B.G., Fariselli L., Tzuk-Shina T., Kortmann R.-D., Carrie C. (2011). Adjuvant Whole-Brain Radiotherapy Versus Observation After Radiosurgery or Surgical Resection of One to Three Cerebral Metastases: Results of the EORTC 22952–26001 Study. J. Clin. Oncol..

[5] Kamp M., Fischer I., Bühner J., Turowski B., Cornelius J.F., Steiger H.-J., Rapp M., Slotty P.J., Sabel M. (2016). 5-ALA fluorescence of cerebral metastases and its impact for the local-in-brain progression. Oncotarget.

[6] Patchell R.A., Tibbs P.A., Regine W.F., Dempsey R.J., Mohiuddin M., Kryscio R.J., Markesbery W.R., Foon K.A., Young B. (1998). Postoperative radiotherapy in the treatment of single metastases to the brain: A randomized trial. JAMA.

[7] Kamp M., Rapp M., Bühner J., Slotty P.J., Reichelt D., Sadat H., Dibué-Adjei M., Steiger H.-J., Turowski B., Sabel M. (2015). Early postoperative magnet resonance tomography after resection of cerebral metastases. Acta Neurochir..

[8] Olesrud I., Schulz M.K., Marcovic L., Kristensen B.W., Pedersen C.B., Kristiansen C., Poulsen F.R. (2019). Early postoperative MRI after resection of brain metastases—Complete tumour resection associated with prolonged survival. Acta Neurochir..

[9] Berghoff A.S., Rajky O., Winkler F., Bartsch R., Furtner J., Hainfellner J.A., Goodman S.L., Weller M., Schittenhelm J., Preusser M. (2013). Invasion patterns in brain metastases of solid cancers. Neuro-Oncology.

[10] Baumert B.G., Rutten I., Dehing-Oberije C., Twijnstra A., Dirx M.J., Debougnoux-Huppertz R.M., Lambin P., Kubat B. (2006). A pathology-based substrate for target definition in radiosurgery of brain metastases. Int. J. Radiat. Oncol..

[11] Preusser M., Capper D., Ilhan-Mutlu A., Berghoff A.S., Birner P., Bartsch R., Marosi C., Zielinski C., Mehta M.P., Winkler F. (2012). Brain metastases: Pathobiology and emerging targeted therapies. Acta Neuropathol..

[12] Berghoff A.S., Preusser M. (2018). Anti-angiogenic therapies in brain metastases. Memo Mag. Eur. Med. Oncol..

[13] Obwegeser A., Jakober R., Kostron H. (1998). Uptake and kinetics of 14C-labelled meta-tetrahydroxyphenylchlorin and 5-aminolaevulinic acid in the C6 rat glioma model. Br. J. Cancer.

[14] Stummer W., Pichlmeier U., Meinel T., Wiestler O.D., Zanella F., Reulen H.-J. (2006). Fluorescence-guided surgery with 5-aminolevulinic acid for resection of malignant glioma: A randomised controlled multicentre phase III trial. Lancet Oncol..

[15] Hadjipanayis C., Widhalm G., Stummer W. (2015). What is the Surgical Benefit of Utilizing 5-Aminolevulinic Acid for Fluorescence-Guided Surgery of Malignant Gliomas?. Neurosurgery.

[16] Kamp M., Grosser P., Felsberg J., Slotty P.J., Steiger H.-J., Reifenberger G., Sabel M. (2011). 5-Aminolevulinic acid (5-ALA)-induced fluorescence in intracerebral metastases: A retrospective study. Acta Neurochir..

[17] Marhold F., Mercea P.A., Scheichel F., Berghoff A.S., Heicappell P., Kiesel B., Mischkulnig M., Borkovec M., Wolfsberger S., Woehrer A. (2020). Detailed analysis of 5-aminolevulinic acid induced fluorescence in different brain metastases at two specialized neurosurgical centers: Experience in 157 cases. J. Neurosurg..

[18] Spanberger T., Berghoff A.S., Dinhof C., Ilhan-Mutlu A., Magerle M., Hutterer M., Pichler J., Woehrer A., Hackl M., Widhalm G. (2012). Extent of peritumoral brain edema correlates with prognosis, tumoral growth pattern, HIF1a expression and angiogenic activity in patients with single brain metastases. Clin. Exp. Metastasis.

[19] Berghoff A.S., Ilhan-Mutlu A., Dinhof C., Magerle M., Hackl M., Widhalm G., Hainfellner J.A., Dieckmann K., Pichler J., Hutterer M. (2014). Differential role of angiogenesis and tumour cell proliferation in brain metastases according to primary tumour type: Analysis of 639 cases. Neuropathol. Appl. Neurobiol..

[20] Bender R., Lange S. (2001). Adjusting for multiple testing—When and how?. J. Clin. Epidemiol..

[21] Siam L., Bleckmann A., Chaung H.-N., Mohr A., Klemm F., Barrantes-Freer A., Blazquez R., Wolff H.A., Lüke F., Rohde V. (2015). The metastatic infiltration at the metastasis/brain parenchyma-interface is very heterogeneous and has a significant impact on survival in a prospective study. Oncotarget.

[22] Schatlo B., Stockhammer F., Barrantes-Freer A., Bleckmann A., Siam L., Pukrop T., Rohde V. (2020). 5-Aminolevulinic Acid Fluorescence Indicates Perilesional Brain Infiltration in Brain Metastases. World Neurosurg. X.

[23] Roberts D.W., Valdés P.A., Harris B.T., Fontaine K.M., Hartov A., Fan X., Ji S., Lollis S.S., Pogue B.W., Leblond F. (2011). Coregistered fluorescence-enhanced tumor resection of malignant glioma: Relationships between δ-aminolevulinic acid-induced protoporphyrin IX fluorescence, magnetic resonance imaging enhancement, and neuropathological parameters: Clinical article. J. Neurosurg..

[24] Kamp M., Munoz-Bendix C., Mijderwijk H.-J., Turowski B., Dibué-Adjei M., Von Saβ C., Cornelius J.F., Steiger H.-J., Rapp M., Sabel M. (2019). Is 5-ALA fluorescence of cerebral metastases a prognostic factor for local recurrence and overall survival?. J. Neuro-Oncol..

[25] Rodewald A., Rushing E.J., Kirschenbaum D., Mangana J., Mittmann C., Moch H., Lugassy C., Barnhill R.L., Mihic-Probst D. (2019). Eight autopsy cases of melanoma brain metastases showing angiotropism and pericytic mimicry. Implications for extravascular migratory metastasis. J. Cutan. Pathol..

[26] Yoo H., Kim Y.Z., Nam B.H., Shin S.H., Yang H.S., Lee J.S., Zo J.I., Lee S.H. (2009). Reduced local recurrence of a single brain metastasis through microscopic total resection. J. Neurosurg..

