# Supplementary material for: Prognostic Value of 5-ALA Fluorescence, Tumor Cell Infiltration and Angiogenesis in the Peritumoral Brain Tissue of Brain Metastases
Source: Cancers (Basel). 2021 Feb 3;13(4):603. doi: 10.3390/cancers13040603 (PMC7913757; doi:10.3390/cancers13040603)
Supplement: Supplementary file 1 [file cancers-13-00603-s001.pdf]

Supplementary Materials

Table S1. Characteristics of multiple resected brain metastases.

		n	%
Number of multiple resected BM		3	(100)
5-ALA fluorescence in BM			
	visible vague fluorescence	3	(100)
5-ALA homogeneity			
	heterogeneous	3	(100)
5-ALA fluorescence in peritumoral brain tissue			
	positive	2	(67)
	negative	1	(33)

5-ALA-5-aminolevulinic acid, BM-brain metastases

Table S2. Univariate and multivariate analyses.

**Time to local recurrence/progression ***								
			**Univariate analyses**			**Multivariate analysis**		
**Variable**	**Category**	***n* (%)**	**HR**	**CI (95%)**	***p*-value**	**HR**	**CI (95%)**	***p*-value**
Angiogenesis		6 (17)	5.924	1.709–20.451	**0.005**	5.607	1.517–20.717	**0.010**
Postoperative treatment								
	None	4 (11)	REF					
	Local radiotherapy	24 (69)	1.249	0.275–5.675	0.774	1.008	0.214–4.746	0.992
	WBRT	7 (20)	2.224	0.350–14.150	0.397	1.232	0.170–8.942	0.837
**One-year Survival**								
			**Univariate analyses**			**Multivariate analysis**		
Angiogenesis		13 (24)	2.228	1.035–4.793	**0.040**	2.629	1.144–6.038	**0.023**
GPA		55 (100)	2.162	1.156–4.043	**0.016**	2.389	1.280–4.456	**0.006**
	Class 1	2 (4)						
	Class 2	8 (15)						
	Class 3	35 (64)						
	Class 4	10 (18)						
Postoperative radiation treatment **								
	None	10 (18)	REF					
	Local radiotherapy	29 (53)	0.782	0.308–2.429	0.782	0.741	0.246–2.227	0.593
	WBRT	15 (27)	1.619	0.559- 4.693	0.375	1.275	0.408–3.986	0.677
	Chemotherapy	21 (38)	0.943	0.442–1.959	0.875	1.156	0.529–2.523	0.716

* Only patients with control MRI (n = 35) included. ** In one patient we did not receive further information about postoperative radiation treatment.
